# Developing Automatic-Labeled Topic Modeling Based on SAO Structure for Technology Analysis

**DOI:** 10.1371/journal.pone.0330275

**Published:** 2025-08-26

**Authors:** Minyoung Park, Sunhye Kim, Byungun Yoon

**Affiliations:** 1 Master student, Department of Industrial & Systems Engineering, School of Engineering, Dongguk University, Seoul, Korea; 2 Post-doctoral researcher, Department of Industrial & Systems Engineering, School of Engineering, Dongguk University, Seoul, Korea; 3 Professor, Department of Industrial & Systems Engineering, School of Engineering, Dongguk University, Seoul, Korea; Universita degli Studi della Campania Luigi Vanvitelli, ITALY

## Abstract

Topic modeling has become essential for identifying emerging technology trends, detecting technological concepts, and forecasting advancements. This study introduces a subject-action-object (SAO) based approach to overcome the limitations of existing auto-labeling methodologies in patent documents. In particular, by utilizing the “Bag of SAO” concept, the study aims to construct topic modeling itself on an SAO basis, thereby clarifying the complex relationships within technology. Traditional auto-labeling methods often lack sufficient quantitative evaluation metrics and overlook the functional significance and hierarchical structure of technologies. To address these challenges, we propose an auto-labeling methodology that combines SAO-based topic modeling and scoring with text summarization and network analysis. The proposed model’s effectiveness was evaluated using the ROUGE score alongside others such as relevance, coverage, and discrimination, showing its ability to capture functional meanings within the technological context. To enhance interpretability, we integrated a hierarchical structure based on CPC subclasses, offering a more comprehensive view of technological development and trends. This approach is expected to improve the accuracy of topic labels while providing deeper semantic insights, contributing to more efficient technology management. This study illustrates how SAO-based auto-labeling methodologies can be applied in the field of technology management, highlighting their potential applications in technology innovation, policy-making, and industry applications. Furthermore, by integrating the SAO structure, this research is anticipated to lay the groundwork for developing more refined methodologies for technology forecasting and diagnosis in future studies. Through this, we hope to gain a clearer understanding of the directions of technological advancement and provide strategic insights for the development of new technologies.

## 1. Introduction

Topic modeling has become an essential method for uncovering future technology trends, recognizing technological concepts, and predicting advancements [1–3]. When applied to patent documents and academic papers, it plays a key role in analyzing research trends, identifying emerging technologies, measuring interdisciplinarity, and enabling technology forecasting. This technique helps bridge the gap between science and technology by capturing contextual concepts through semantic analysis, addressing challenges like synonyms and polysemy [2,4,5].

Accurate topic labeling is critical for making the results of topic modeling understandable and actionable. This labeling process simplifies interpretation by connecting the word probability distributions within topics to intuitive, relevant information about the technology [6]. Since topic modeling is widely used in knowledge management across various fields, precise interpretation and labeling are essential to reflect the true meaning of each topic. As a result, topic labeling has gained prominence as an interpretive method. Traditionally, researchers have manually labeled topics by interpreting the meaning of related words, but this approach is time-consuming and prone to inconsistencies due to differences in domain-specific knowledge. To overcome these issues, several studies have introduced auto-labeling techniques [7–10]. Early auto-labeling efforts were based on probabilistic methods but faced limitations, as they were typically validated through qualitative comparisons with expert-labeled topics, lacking quantitative metrics to assess whether the automatically generated labels were more appropriate than those assigned by experts. Additionally, these approaches did not clearly define the technological level the labels should reflect, nor did they account for the hierarchy between labels, leading to inconsistencies in label levels. Therefore, a technology-specialized methodology for automatic topic labeling that consistently captures the functional aspects of technologies is yet to be established.

This study aims to overcome the limitations of previous research in topic modeling, specifically focusing on Latent Dirichlet Allocation (LDA), by incorporating the subject-action-object (SAO) structure in the auto-labeling process. LDA has become a prevalent method for uncovering patterns within textual data; however, it often encounters challenges in accurately representing the relationships between technological concepts. The SAO semantic analysis method examines the relationships expressed through actions between subjects and objects in sentences, allowing for a more nuanced understanding of technological relationships. By applying this structure, the study can more effectively extract technological information from each sentence and document [11]. Utilizing the SAO structure helps highlight the “core concepts” of technological contents in patents and provides deeper insights through the semantic relationships between these concepts [12]. Given that patent documents are rich in technological information, applying the SAO-based approach enables a comprehensive analysis of these contents. Furthermore, traditional methods often fail to capture the hierarchical nature of technology, which is crucial for understanding its evolution. This study aims to enhance the accuracy of labels and establish a robust framework that reflects the functional significance and interrelationships of technologies within the patent ecosystem by integratively applying the Bag of SAO concept throughout the topic modeling and labeling processes. This approach underscores the necessity for more sophisticated analytical methods in light of the increasing complexity of technological advancements. The need for an LDA model that utilizes the SAO structure, along with effective topic labeling methods, can be emphasized from several perspectives. First, traditional LDA models have limitations in capturing the relationships between words, making it difficult to clearly delineate the roles of words within context. By introducing the SAO structure, the interactions and relationships among various components of technology can be expressed more clearly, which is essential for understanding the context of technological advancements and strengthening the connections between topics. Second, effective topic labeling is critical for making the results of topic modeling understandable and actionable. The SAO-based approach enhances the labeling process by clearly defining the roles of subjects, actions, and objects within each topic. This allows researchers to create labels that accurately reflect the underlying technological relationships, moving beyond mere word frequency or co-occurrence to capture the intrinsic functions and interactions of technologies. Third, the SAO-based LDA can more effectively reflect the hierarchical structure of technology. As technology evolves, various levels of technological concepts exist, and the SAO structure can distinctly reveal these hierarchical relationships. This capability is crucial not only for analyzing technological progress but also for generating labels that encapsulate the functional significance of technologies at different levels.

Therefore, the SAO-based LDA offers a comprehensive methodology for understanding the complexities of technology, clarifying the relationships between technologies, and making research findings more practically applicable. This study aims to propose a framework specialized for technology management through the integration of the SAO structure with the LDA and topic labeling approaches, thereby establishing the following specific objectives:

- **Identify meaningful labels** in technological development and industry and propose an auto-labeling methodology for topic modeling that integrates text summarization and network analysis.
- **Structure topic models and labels** using the SAO framework to develop a topic modeling approach specialized for technology management.
- **Interpret topics** by incorporating patent CPC codes to reflect the hierarchical nature of technology.

The paper has been structured as follows. Section 2 reviews existing methodologies and the application of the SAO structure, including SAO-based topic modeling for auto labeling, in the field of topic modeling. Section 3 introduces the SAO-based auto-labeling methodology, integrating text summarization and network analysis. Section 4 presents the application of this methodology, including quantitative and qualitative evaluations. Section 5 summarizes the research methodology and results, discussing contributions and improvements. Finally, Section 6 concludes with a discussion of the study’s findings.

## 2. Related work

### 2.1. Automatic labeling of topic models

Topic modeling is a technique used to uncover hidden themes in large text corpora. Typically, topics are labeled using the top “n” terms with the highest marginal probability values [13,14]. While this method helps identify main topics, the terms generated by the algorithm may not always align with user perspectives, leading to inconsistent labeling. To address this issue, foundational methodologies have been proposed for automatic topic labeling. Early work by [7], approached automated labeling as an optimization problem. Their probabilistic method aimed to maximize mutual information between words and labels while minimizing Kullback-Leibler divergence between word distributions. With the introduction of word embeddings, subsequent studies enhanced this probabilistic approach by incorporating word vectors [15–17]. One approach involved using a chunk parser to generate candidate labels, which were then mapped to word vectors and letter trigram vectors. This method derives semantic associations based on the similarity between candidate labels and topics [17]. Other studies have explored topic modeling and labeling by considering the hierarchical nature of technologies [18,19]. For instance, ontology analysis-based auto-labeling methodologies leverage hierarchical relationships between topics to improve labeling accuracy and facilitate interpretation [20]. Additionally, some methodologies have applied text summarization techniques to label topics. [8] proposed extracting sentences related to topics and labeling them at various levels—word, phrase, and sentence. [21] introduced a topic-labeling method using summarization algorithms from sources other than Twitter, which provided labels for LDA results. Another method, the sum basic algorithm, calculates document weights, selects high-weight words, and uses them to generate labels [21]. Overall, numerous studies have explored text summarization to enhance the labeling of topic modeling results.

Among the methods developed for auto-labeling are phrase-level labeling, hierarchical labeling through ontology analysis, and labeling using text summarization techniques. This study aims to advance auto labeling for topic models by employing SAO structure to capture the functional semantic relationships of technologies and integrating network analysis to explore the connections between SAOs. Specifically, we propose an enhanced auto-labeling methodology for LDA [22], a widely used topic modeling technique. Our approach focuses on reflecting both the structural and functional characteristics of technology, offering a distinct improvement over existing methods that primarily emphasize word-level labeling.

### 2.2. SAO structure and SAO-based topic modeling

SAO semantic analysis examines the relationship between subjects and objects in a sentence, mediated through actions. This analysis can be framed in a problem & solution (P&S) format, where subjects represent the “solution,” actions denote the “effect” or “impact” of the solution, and objects signify the “invention problem.” This structure has been demonstrated as an effective tool for problem-solution pattern analysis [23–25].

**Fig 1:**
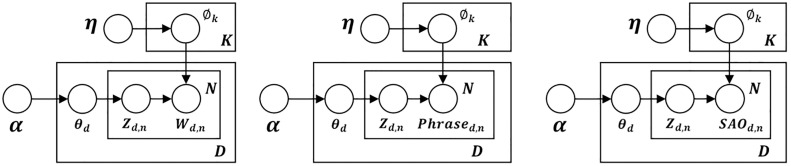
Graphical models of LDA: (a) word, (b) phrase, and (c) SAO-based technological topic model. (D: number of documents; K”: number of topics; and N: number of unique words/phrases/SAOs).

Utilizing SAO structures to characterize patent content offers distinct advantages over traditional methods. Research has explored integrating SAO analysis with topic modeling to uncover problem and solution patterns in technical documents [26]. This study addresses the limitations of word-level LDA in analyzing technical context for competitive technical intelligence (CTI). It employs SAO structures to enhance LDA analysis, focusing on problem and solution patterns. The approach extends the bag of word model to the bag of SAOs, as shown in Fig 1 (c). This generates a D×N document-SAO matrix ${SAO}_{D\times N}$ for a patent corpus, where D represents documents and *N* denotes $\;{SAO}_{s}$.

This matrix is represented by ${SAO}_{D,N}$ = $freq({SAO}_{t}=n|d_{t}=d)$, showing the distribution of ${SAO}_{s}$ based on the probability matrix ϕ, as derived from the conditional distribution $\emptyset_{k,i}=p({SAO}_{t}=i|z_{t}=k)$, which involves K × N parameters. The combined probability of SAO and latent topics for the target patent corpus is calculated as follows:


$$P\left(SAO,z|\phi ,\theta \right)=\prod_{i=1}^{N}\prod_{k=1}^{K}\prod_{d=1}^{D}\emptyset_{k,i}^{N_{k,i}}\theta_{d,k}^{N_{d,k}}.$$ (1)

Here, EM-based variable inference is performed to obtain the probability matrices ϕ and θ.

Through this approach, we confirm that SAO-based LDA, which utilizes the bag of SAO concept, is mathematically valid. The SAO structure offers a more detailed description of topics by highlighting Problem & Solution Patterns, making it more effective than traditional word-level LDA. In this study, we propose a technology management-specific topic modeling and auto-labeling methodology based on SAO-based LDA. This method incorporates text summarization to generate labels within the SAO structure. The expected outcome is more meaningful topic labels, enabling quicker topic comprehension and more efficient information navigation.

## 3. Proposed framework

This study presents an auto-labeling topic modeling methodology designed to capture the functional aspects of technology using the SAO structure. The research focuses on developing a methodology that identifies meaningful labels in technology development and derives an auto-labeling method applicable to topic modeling through text summarization and network analysis. By constructing a topic model and corresponding labels using the SAO framework, the method captures more detailed functional meaning information about technologies. Additionally, after modeling, patent CPC codes are used in the qualitative analysis to reflect the hierarchical structures of technologies.

### 3.1. Overall process

**Fig 2:**
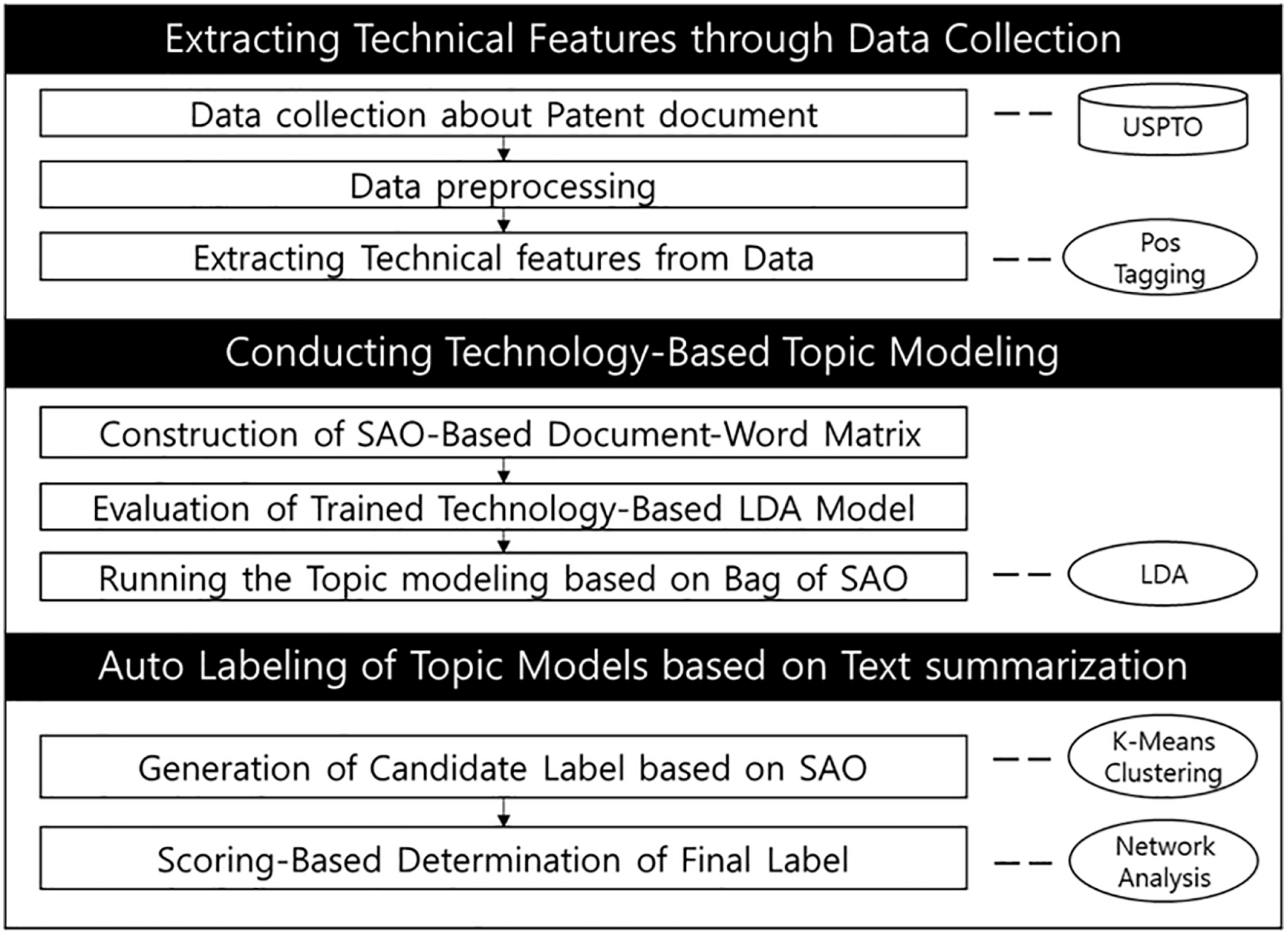
Overall framework.

The proposed methodology consists of three modules: Extracting technical features through data collection, conducting technology-based topic modeling, and automatically labeling topics based on text summarization. As shown in Fig 2, the framework begins by extracting technical features from patent documents, identifying key topics and key SAOs through SAO-based topic modeling, and labeling them accordingly. This process enables researchers to identify key technical features, trends, and topics within a given domain, providing an efficient method to quickly comprehend and navigate the information.

### 3.2. Extracting technical features through data collection

**Fig 3:**
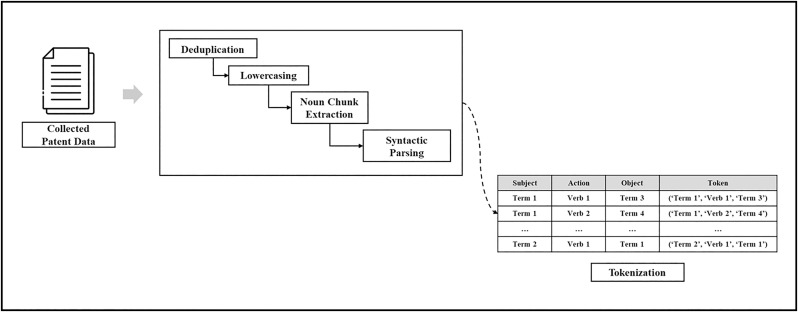
Illustration of extracting technical features through data collection.

For data collection, patent documents are sourced from the USPTO database using specific keywords. The collected data include patent numbers, summaries, and CPC subclasses. During preprocessing, documents are broken down into sentence-level units, and stopwords such as special characters, articles, and particles are removed. Additional preprocessing steps include converting text to lowercase, extracting noun chunks, and performing syntactic parsing. The technical features are then extracted using the spaCy (en_core_web_sm) model, specifically focusing on SAO structures, which are treated as primary units of analysis. The process for extracting these features is demonstrated in Fig 3.

### 3.3. Conducting technology-based topic modeling

#### 3.3.1 Construction of SAO-based document-word matrix.

To construct the SAO-based document-word matrix, we input the extracted SAO structures into the LDA model and build a document-word matrix. This process utilizes the “CountVectorizer” from the scikit-learn library, which creates a bag of SAO representations. In this matrix, each row represents a document (SAO tuple) and each column represents a unique word (SAO structure).

#### 3.3.2. Running the topic modeling based on the bag of SAO.

To derive SAO-based LDA results, initialize the LDA model with the specified number of topics (=T) using “LatentDirichletAllocation” from scikit-learn. We use quantitative metrics such as perplexity and coherence scores to select the appropriate number of topics. These metrics are representative of LDA topic allocation and are statistical modeling techniques used as a posteriori. Among machine learning techniques, this probabilist approach models the number of latent topics (T) in a document based on unsupervised learning [27]. This posterior evaluation of model stability allows for determining the number of topics based on data rather than a priori judgments. Perplexity measures entropy, with a lower value indicating a more stable organization of topics and less disorder. To find the number of stable topics, we need to identify the number of topics with low perplexity. The calculation process is shown in Equation (2):


$$Perplexity\left(D|\theta ,\beta \right)=𝐞𝐱𝐩\left(-\frac{\sum_{d=1}^{M}𝐥𝐨𝐠p\left(d|\theta ,\beta \right)}{\sum_{d=1}^{M}N_{d}}\right),$$ (2)

where D is the entire document; M is the number of documents; N_d is the number of words in document d; p(d|θ,β) is the likelihood of the model given document d, where θ is the distribution of topics and β is the distribution of bag of ${SAO}_{s}$.

As the number of topics T increases in a corpus of M documents, the number of words assigned to each topic decreases, resulting in a decrease in perplexity. While this metric enables quantitative performance evaluation of topic modeling, it has the limitation of showing a decreasing trend as T increases, theoretically reaching its lowest value when the number of topics equals the number of documents [28].

To address this limitation, we also considered the coherence score in our study. This metric evaluates the degree of semantic connection between words, with a higher score indicating a better connection. The coherence score for a given set of words is calculated using Equation (3) [29,30]. The numerator in Equation (3) increases with the co-occurrence of two words within a document, enhancing coherence. Let $\left|\left\{s_{i},s_{j}\right\}\right|{\;s}_{i}\neq s_{j}\}|$denote the number of all possible SAO pairs, and $sim(s_{i},s_{j})$ represents the semantic similarity of the SAO pair $\left(s_{i},s_{j}\right)$:


$$Coherence\text{\textbackslash{} }Score=\frac{2}{\left|\left\{s_{i}\text{,}s_{j}\right\}\right|{\text{\textbackslash{} }s}_{i}\neq s_{j}\}|}\sum_{\text{\textbackslash{} }s_{i}\neq s_{j}}sim(s_{i}\text{,}s_{j}).$$ (3)

In this study, we used both perplexity and coherence score metrics to determine the optimal number of topics T for better result interpretation, thus validating the stability of the LDA model. Based on these values, we trained the LDA model using the SAO document-word matrix (sao_matrix). SAO tuples were grouped by assigned topics, necessitating adjustments to the LDA hyperparameters. Key hyperparameters for the LDA model, which serve as benchmarks for result derivation, include the optimal number of topics T, and the parameters α and β [31,32]. There are no fixed absolute values for these parameters; instead, a range of values is explored through multiple iterations to determine the optimal settings based on perplexity and ease of result interpretation [33]. To facilitate ease of interpretation, we examined whether keywords clustered around a single topic and adjusted the hyperparameters T, α, and β\ based on both perplexity results and interpretability to achieve the outcomes.

### 3.4. Auto labeling of topic models based on text summarization

#### 3.4.1 Generation of candidate labels based on SAO.

In this process, auto-labeling for each topic was performed using the SAO structure. To apply text summarization to the top SAO structures of each topic, the study reviewed a word embedding-based text summarization technique. The algorithm employed corresponds to an unsupervised learning-based automatic summarizer that preserves the semantic information of the text during summarization [34]. The semantic-based summarization reduces redundancy in the input data while maintaining meaning, highlighting its efficiency and usefulness in the labeling process. Therefore, the study aims to use the SAO structures extracted from the summarized text generated by the algorithm and modify them for topic labeling. The process is as follows: each SAO structure of a topic is tokenized and converted into vector representations using Word2Vec. K-means clustering is then applied to group documents into a specified number of clusters. Based on the distance between the center of each cluster and each sentence, representative sentences from the closest cluster are identified and merged to create a summary document. Instead of simply applying the existing text summarization algorithm, we extend its contribution by modifying it and applying a separate process to re-extract the SAO structure for the derived summary. To evaluate the appropriateness of the label assignment results for each topic, we calculated the Silhouette score for K-means clustering and determined the optimal number of clusters (K) for each topic. The Silhouette score measures clustering quality by assessing the degree of cohesion and separation within the clustering results [35]. In this study, by calculating the Silhouette score for each topic, we aim to evaluate reliability by considering how well the topic clusters together and how distinctly it separates from other topics. This approach allows us to extract representative sentences from each topic based on the optimal k value and summarize them to produce descriptive results. We then derive k candidate topic labels based on the appropriate k value for each topic and propose a method to confirm the representative label by scoring the candidate labels. The final algorithm for the candidate topic label derivation process is shown in Algorithm 1.

Algorithm 1: Text summarization algorithm for candidate label generation based on SAO structure

Result: SAO-structured candidate label based on summarized document

Input: SAO distribution(s)

Output: Summarized document (SD), SAO-structured candidate labels

Let s be the SAO distribution representing the input document

For each sentence $s_{i}$ in the SAO distribution s:

 Tokenize ($s_{i}$) to obtain $W=\{w_{1},w_{2},\;$*…* $,w_{m}\}$

 For each word $w_{j}$ in W:

  Convert $w_{j}\;$to its vector representation${\;V}_{j}$ using Word2Vec.

 Concatenate the vectors ${\;V}_{1},$ ${\;V}_{2},\;\ldots \;,$ ${\;V}_{\left|w\right|}$ to obtain ${BV}_{i}$, the sentence vector.

Let BV = $\left\{{BV}_{1},\;{BV}_{2}\text{, }\ldots \;,\;{BV}_{n}\right\}$ be the collection of sentence vectors for all sentences in S.

Perform K-Means clustering on ${BV}_{i},\;$to obtain k_clusters.

Compute the Silhouette Score for each clustering to determine the optimal *k*.

Select the clustering with the highest Silhouette Score as *k_optimal*.

Rank the *k_optimal* clusters based on their distances to the centroid.

Select representative sentences from each cluster to form the summarized document SD.

For each selected sentence $s_{i}$ in SD:

  Extract SAO triples and add them to a list of SAO-based candidate labels.

Return the summarized document SD and the SAO-structured candidate labels as the final result.

#### 3.4.2 Scoring-based determination of final label.

The final label is derived based on the scoring of the previously established candidate labels. In this process, we utilize a scoring method that converts centrality values through network analysis for each topic. Network analysis quantitatively examines the structure of relationships between objects by representing them as a network [36]. The objects under analysis are modeled as nodes, with the connections between them represented as edges (links). The centrality indicator helps identify which nodes are crucial for understanding the relationships among individual nodes [37]. In this study, we aim to construct a network based on SAO units for each topic. The nodes consist of SAO units themselves (${SAO}_{1}$, ${SAO}_{2}$,...) and the candidate labels (${Label}_{1}$, ${Label}_{2}$,...), while the edges represent connections between SAO units derived from the same document. An example of this identification is shown in Table 1.

**Table 1:** Example of identifying the same document (Patent No.).

Topic #	(‘S’, ‘A’, ‘O’)	Patent No.
Topic 1	(‘data values’, ‘use’, ‘machine learning models’)	US11418523
	(‘clustering module’, ‘cluster’, ‘data values’)	
	(‘order’, ‘deliver’, ‘end solution’)	US11120365
	(‘AI’, ‘respond’, ‘voice’)	US11120365
	(‘AI’, ‘respond’, ‘other commands’)	
	(‘furniture assembly’, ‘include’, ‘speaker’)	
	…	…
Topic 2	(‘memory’, ‘store’, ‘instructions’)	US10825205
	(‘image’, ‘comprise’, ‘parameter’)	
	(‘AI’, ‘provide’, ‘numerous functions’)	US10979241
	…	…
…	…	…

**Fig 4:**
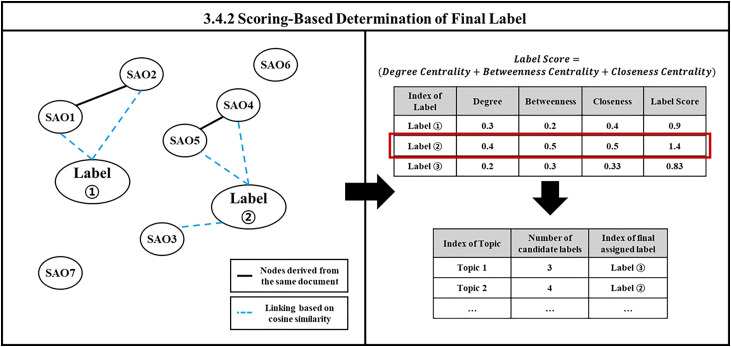
Illustration of scoring-based determination of final label.


We incorporate the concept of the bag of SAO into LDA itself [26], which supports the decision to use SAO units as nodes in the network. Since the candidate labels follow the process outlined in Algorithm 1, it is challenging to directly depict their relationships. To address this, we construct a network graph by linking SAO units that are determined to be similar based on cosine similarity values (threshold = 0.5). The centrality of the candidate label nodes is then calculated to evaluate their importance (label score), and the centrality scores of each component are summed up to form the final label score. This network analysis calculates the centrality of each SAO unit and candidate label for each topic, allowing us to assign the final label based on the label score. This score identifies the label that represents the functional meaning of each topic. The final considered label score is shown in Equation (4), and the entire process is illustrated in Fig 4. The description of the algorithm is shown in Algorithm 2.

Label Score = (Degree Centrality + Betweenness Centrality + Closeness Centrality)  (4)

Algorithm 2: Scoring-based determination of final label

Result: Final label for each topic based on SAO units and candidate labels

Input: Graph *G* representing SAO structures and candidate labels

Output: Final label for each topic, Centrality scores table

Function create_graph_for_topic(topic_saos, candidate_labels, topic):

 Initialize an empty graph G

 For each (subject, verb, object_) with score in topic_saos:

  Add node (subject, verb, object_) to G with score attribute

 For each label in candidate_labels:

  Add node label to G with score attribute

 For each (subject, verb, object_) with score in topic_saos:

  For each label in candidate_labels:

   ompute cosine similarity score between (subject, verb, object_) and label

   If cosine similarity score >= threshold:

    Add edge between (subject, verb, object_) and label to G with similarity attribute

Function calculate_centrality_and_determine_label(G, candidate_labels):

 Compute centrality (Degree, Betweenness, Closeness) for nodes in G

 Compute total score for each candidate label by summing up centrality scores

 Determine final label as the one with the highest total score

 Generate centrality scores table including centrality (Degree, Betweenness, Closeness) and total score for each candidate label

For each topic, saos in topic_data:

 Create graph G for topic using SAO structures and candidate labels

 Calculate centrality scores and determine final label for topic using G and candidate labels

 Output final label and centrality scores table for the topic

## 4. Results

### 4.1. Data acquisition and feature extraction process

For data collection, we gathered patent documents registered with the USPTO accessible through the advanced search page of the patent public search website (https://www.uspto.gov/patents/search/patent-public-search) between 2018 and 2023 to analyze recent research trends. Two technology domains were selected to demonstrate the applicability of the proposed approach. The first domain, Artificial Intelligence (AI), has shown rapid technological advancement in recent years, with active research and frequent patent filings. The second domain, Biotechnology, was chosen due to its stable growth and ongoing integration across various industries, accompanied by continuous technological development. Patent data were retrieved from the database of Wisdomain, a patent analysis platform using the keywords “Artificial Intelligence” and “Biotechnology,” with a focus on the CPC classifications [H04: ELECTRIC COMMUNICATION TECHNIQUE] for AI and [A61: MEDICAL OR VETERINARY SCIENCE; HYGIENE] for Biotechnology. As a result, we obtained 700 patents for AI and 2,579 patents for Biotechnology (https://github.com/minongs/Automatic-Labeled-Topic-Modeling-Based-on-SAO-Structure) The collected data included patent numbers, abstracts, and CPC classifications. For data preprocessing, we performed sentence-level segmentation and removed stop words, special characters, articles, and particles. To extract technological features, we employed the spaCy (*en_core_web_sm*) model. As a result, we extracted 3,209 Subject-Action-Object (SAO) structures for the AI domain and 10,759 SAO structures for the Biotechnology domain, which were used for further analysis.

### 4.2. Implementation of technology-based topic modeling

**Fig 5:**
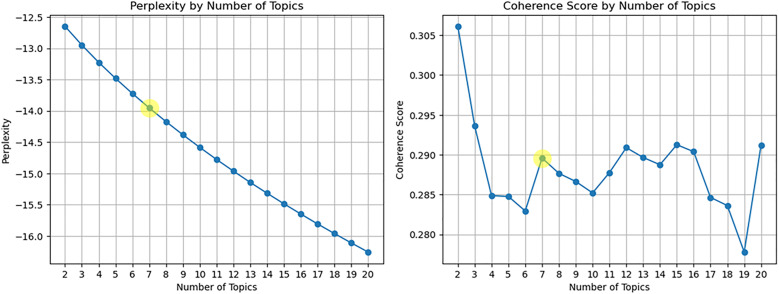
Evaluation of trained SAO-based LDA model (Perplexity, Coherence score) (AI).

**Fig 6:**
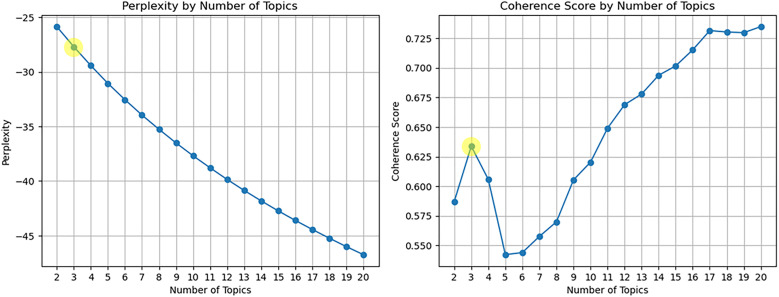
Evaluation of trained SAO-based LDA model (Perplexity, Coherence score) (Biotechnology).

Next, we constructed the SAO-based document-word matrix and evaluated the performance of the trained SAO-based LDA model. As shown in Figs 5 and 6, the optimal number of topics was determined by selecting configurations with low perplexity and high coherence scores, ensuring the model’s stability. Specifically, the optimal number of topics was identified as $T_{1}$ =7 for the Artificial Intelligence domain and $T_{2}$= 3 for the Biotechnology domain.

Based on these findings, we conducted topic modeling using the bag-of-SAO approach, where extracted SAO tokens were used as input for the LDA model. The final hyperparameter settings, determined by balancing perplexity results and interpretability, are summarized in Table 2.

**Table 2:** Hyperparameter Settings for SAO-Based LDA Model by Technology Domain.

Technology Domain	Number of Topics ($T_{i}$)	Minimum collection frequency (words & documents)	Alpha (α)	Beta (β)	Iteration
AI	7	5	1	0.01	1,000
Biotechnology	3	5	1	0.01	1,000

*Note:* α controls the document-topic distribution, and β controls the topic-word distribution. Both are Dirichlet priors.

The outputs for both domains included (1) the distribution of SAO structures across topics and (2) the distribution level of SAO tokens per topic.

### 4.3. Automated topic labeling through text summarization and network analysis

**Fig 7:**
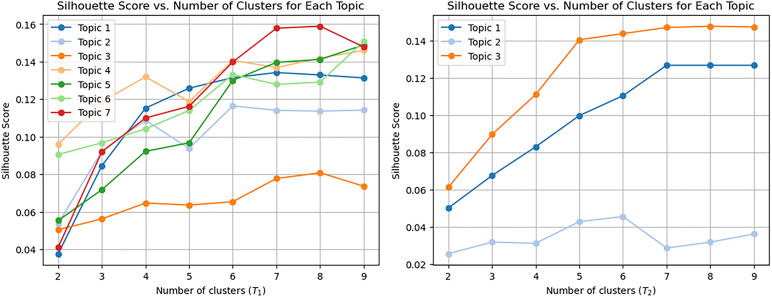
Silhouette Score vs. Number or Clusters for Each Topic (${𝐓}_{1}$: AI, ${𝐓}_{2}$: Biotechnology).

To generate candidate labels using SAO structures, we conducted text summarization. During this process, we verified the representativeness of the labels by determining the optimal number of clusters (K) for K-means clustering, using the Silhouette Score, as illustrated in Fig 7 and Tables 3 and 4. The calculated K values were then applied to each topic accordingly.

**Table 3:** Results of deriving the optimal K-value by topic (AI).

Topic #	Optimal K-value	Topic #	Optimal K-value
#1	3	#5	6
#2	4	#6	6
#3	4	#7	6
#4	4	–	–

**Table 4:** Results of deriving the optimal K-value by topic (Biotechnology).

Topic #	Optimal K-value	Topic #	Optimal K-value
#1	7	#3	5
#2	6	–	–

**Fig 8:**
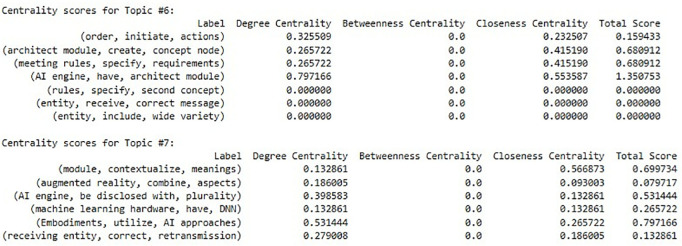
Example of candidate label scoring (AI).

This approach enabled us to derive SAO-level labels that reflect the distribution of SAO structures within each topic. Subsequently, we scored the candidate labels and converted these scores to finalize the most representative label for each topic. The label scoring results are presented in Fig 8, which visualizes the relative scores of the candidate labels across topics.

**Fig 9:**
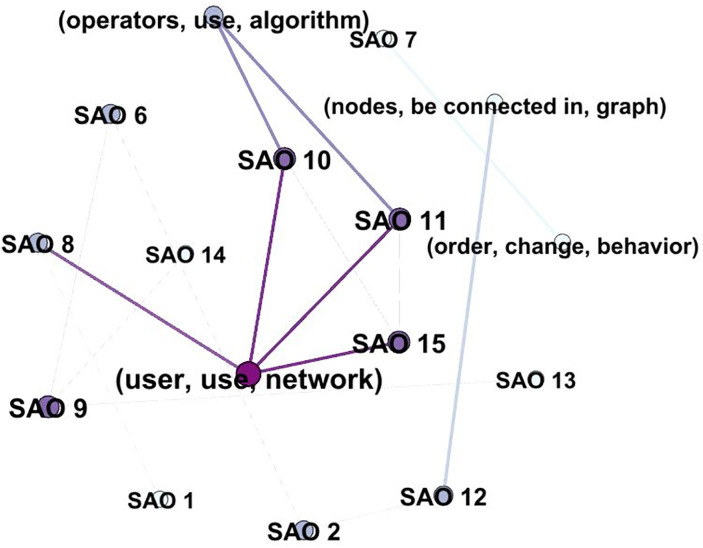
Example network graph of the candidate label selection process (AI).

Ultimately, the label with the highest total score was selected as the final label. An example visualization of this process is shown in Fig 9 where SAO nodes derived from the same document are connected, and edges are established based on cosine similarity to the candidate labels.

The candidate labels and the final assigned labels for each topic are presented in Tables 5 and 6. The appropriateness of the SAO units and the final assigned labels for each topic is discussed in Section 4.5, “Evaluation through Qualitative Interpretation.” The complete results of the LDA modeling and labeling process are provided in Appendix 1 for the artificial intelligence domain and Appendix 2 for the biotechnology domain.

**Table 5:** Candidate label information and final assigned label by topic (AI).

Label # Topic #	Label 1	Label 2	Label 3	Label 4	Label 5	Label 6
Topic 1	(determination, exceed, parameter)	**(AI,** **respond, plurality**)	(plurality, set, information)	–	–	–
Topic 2	(AI engine, include, learner)	**(machine learning, enhance, hardware performance)**	(second parameter, exceed, first parameter)	(AI, provide, numerous functions)	–	–
Topic 3	(order, change, behavior)	(nodes, be connected in, graph)	(operators, use, algorithm)	**(user,** **use, network)**	–	–
Topic 4	**(AI engine,** **include,** **learner module)**	(system, include, learning engine)	(intelligent umbrella, include, microphones)	(component, send, device)	–	–
Topic 5	(cyber, threat, coordinator)	(AI engine, include, instructor module)	**(component,** **identify,** **devices)**	(networks, increase, latency)	(tasks, include, wide variety)	(component, display, input data)
Topic 6	(order, initiate, actions)	**(AI engine,** **have,** **architect module)**	(architect module, create, concept node)	(meeting rules, specify, requirements)	(entity, correct, faulted message)	(entity, receive, correct message)
Topic 7	(module, contextualize, meanings)	(augmented reality, combine, aspects)	(AI engine, be disclosed with, plurality)	(machine learning hardware, have, DNN)	**(Embodiments, utilize,** **AI approaches)**	(receiving entity, correct, retransmission)

**Table 6:** Candidate label information and final assigned label by topic (Biotechnology).

Label # Topic #	Label 1	Label 2	Label 3	Label 4	Label 5	Label 6	Label 7
Topic 1	(‘IVI’, ‘be inserted in’, ‘opposite position’)	**(‘present invention’, ‘provide’, ‘method’)**	(‘light’, ‘be directed toward’, ‘tip’)	(‘IVIs spectrometer’, ‘include’, ‘plurality’)	(‘opposite position’, ‘interconnect’, ‘member stabilize’)	(‘analyte measurer’, ‘measure’, ‘method’)	(‘spectrometer’, ‘include’, ‘unit’)
Topic 2	**(‘measurement device’, ‘analyze’, ‘biological sample’)**	(‘blood’, ‘be expressed in’, ‘biological sample ‘)]	(‘method’, ‘determine’, ‘treatment’)	(‘gene’, ‘be expressed in’, ‘biological sample’)	(‘treatment measurement device’, ‘include’, ‘sensor delivery level’)	(‘recorder’, ‘record’, ‘gene’)	–
Topic 3	(‘delivery’, ‘include’, ‘damage’)	(‘delivery’, ‘include’, ‘stretchable sensor’)	(‘MP’, ‘transmit’, ‘control’)	(‘MP’, ‘transmit’, ‘reagent’)	**(‘module’, ‘record’, ‘control signal’)**	–	–

### 4.4. Quantitative evaluation

For the quantitative evaluation of the results presented in Section 4.3, we utilized the recall-oriented understudy of gisting evaluation (ROUGE) score along with the metrics of relevance, coverage, and discrimination from existing studies.

1) Using the ROUGE score to evaluate text summarization performance

ROUGE is a method used to compare and evaluate automatically summarized documents against those manually summarized by experts [38]. ROUGE-N employs n-grams to calculate recall and precision, ultimately providing an F-score as the final result. The types of ROUGE scores include ROUGE-1, ROUGE-2, and ROUGE-L, which measure unigram, bigram, and the longest matching subsequence, respectively. In this study, the full text of the original summary served as the reference data, and the performance of the proposed method was compared with those of existing auto-labeling results [7] and other summarization models (OPINOSIS, PageRank). The equation for calculating the ROUGE score is shown:


$$ROUGE-N=\frac{\sum_{S\in \left[ReferenceSUmmary.\right]}\sum_{gram_{n}\in S}Count_{match}\left(gram_{n}\right)}{\sum_{S\in \left[ReferenceSUmmary.\right]}\sum_{gram_{n}\in S}Count\left(gram_{n}\right)}.$$ (5)

The final evaluation results are presented in Tables 7 and 8, confirming that the proposed model consistently outperformed existing methods across both technology domains—AI and biotechnology. In the AI domain, the proposed model achieved the highest ROUGE-1 score overall, indicating its strong ability to capture the core content of the documents. For ROUGE-2, the average scores were as follows: [7] at 0.0128, OPINOSIS at 0.2682, PageRank at 0.2707, and the proposed model at 0.298, making the proposed model the best performer. This demonstrates that the proposed model excels in capturing the relationships between words and their contextual meaning.

**Table 7:** Rouge score evaluation results for comparing summarization outcomes (AI).

	Topic 1		Topic 2		Topic 3		Topic 4		Topic 5		Topic 6		Topic 7	
[7]	Rouge1	0.0714	Rouge1	0.0988	Rouge1	0.0851	Rouge1	0.0899	Rouge1	0.1136	Rouge1	0.0789	Rouge1	0.0833
	Rouge2	0.0000	Rouge2	0.0000	Rouge2	0.0435	Rouge2	0.0000	Rouge2	0.0465	Rouge2	0.0000	Rouge2	0.0000
	RougeL	0.0476	RougeL	0.0741	RougeL	0.0638	RougeL	0.0674	RougeL	0.1136	RougeL	0.0526	RougeL	0.0625
OPINOSIS	**Rouge1**	**0.3505**	Rouge1	0.3441	Rouge1	0.2692	Rouge1	0.3333	Rouge1	0.3564	Rouge1	0.3820	Rouge1	0.1284
	**Rouge2**	**0.2947**	Rouge2	0.2857	Rouge2	0.2157	Rouge2	0.3200	Rouge2	0.3232	Rouge2	0.3448	Rouge2	0.0935
	**RougeL**	**0.3505**	**RougeL**	**0.3441**	RougeL	0.2692	**RougeL**	**0.3333**	**RougeL**	**0.3564**	RougeL	0.3820	**RougeL**	**0.1284**
PageRank	Rouge1	0.3505	Rouge1	0.3441	Rouge1	0.2857	Rouge1	0.3333	Rouge1	0.3564	Rouge1	0.3820	Rouge1	0.1284
	Rouge2	0.2947	Rouge2	0.2857	Rouge2	0.2330	Rouge2	0.3200	Rouge2	0.3232	Rouge2	0.3448	Rouge2	0.0935
	RougeL	0.3505	**RougeL**	**0.3441**	**RougeL**	**0.2857**	**RougeL**	**0.3333**	**RougeL**	**0.3564**	**RougeL**	**0.3820**	**RougeL**	**0.1284**
Proposed	Rouge1	0.2609	**Rouge1**	**0.4124**	**Rouge1**	**0.3333**	**Rouge1**	**0.4259**	**Rouge1**	**0.5179**	**Rouge1**	**0.5149**	**Rouge1**	**0.1864**
	Rouge2	0.2000	**Rouge2**	**0.3579**	**Rouge2**	**0.2642**	**Rouge2**	**0.3585**	**Rouge2**	**0.4182**	**Rouge2**	**0.3838**	**Rouge2**	**0.1034**
	RougeL	0.1739	RougeL	0.2062	RougeL	0.2593	RougeL	0.3148	RougeL	0.2857	RougeL	0.3564	RougeL	0.1017

**Table 8:** Rouge score evaluation results for comparing summarization outcomes (Biotechnology).

	Topic 1		Topic 2		Topic 3	
[7]	Rouge1	0.1235	Rouge1	0.0217	Rouge1	0.0430
	Rouge2	0.0253	Rouge2	0.0000	Rouge2	0.0000
	RougeL	0.0741	RougeL	0.0217	RougeL	0.0430
OPINOSIS	Rouge1	0.1818	Rouge1	0.1064	Rouge1	0.2083
	Rouge2	0.0206	Rouge2	0.0217	Rouge2	0.1200
	RougeL	0.0606	RougeL	0.0638	**RougeL**	**0.1961**
PageRank	Rouge1	0.2947	Rouge1	0.3178	Rouge1	0.2424
	Rouge2	0.2151	Rouge2	0.2476	**Rouge2**	**0.1856**
	RougeL	0.2526	RougeL	0.2991	RougeL	0.1616
Proposed	**Rouge1**	**0.4950**	**Rouge1**	**0.4071**	**Rouge1**	**0.2549**
	**Rouge2**	**0.3619**	**Rouge2**	**0.2703**	Rouge2	0.1200
	**RougeL**	**0.2804**	**RougeL**	**0.3363**	**RougeL**	**0.1961**

In the case of ROUGE-L, OPINOSIS and PageRank yielded relatively higher scores, which can be attributed to the characteristics of their algorithms. OPINOSIS generates summaries by evaluating sentence-level similarity and therefore performs well in capturing diverse contexts. PageRank, originally developed to assess webpage importance, constructs summaries based on the network structure of significant sentences or phrases in the document, resulting in high ROUGE-L scores from a global structural perspective. In contrast, the proposed model, which builds summaries based on SAO structures that emphasize functional and relational meaning, focuses more on technical salience and core content rather than inter-sentence connectivity. Nevertheless, the proposed model still maintained a reasonable level of performance in the ROUGE-L metric, while achieving particularly strong improvements in ROUGE-1 and ROUGE-2. In the biotechnology domain, the proposed model showed superior performance across all metrics—ROUGE-1, ROUGE-2, and ROUGE-L—except in Topic 3, where its ROUGE-2 score was slightly lower than that of PageRank. Despite the complexity and high concentration of technical terms in documents within this domain, the SAO-based approach effectively captured semantic units and summarized the context. Notably, the proposed model even outperformed OPINOSIS and PageRank in ROUGE-L, providing strong evidence of its capability to handle the overall structure and essential information in technical documents.

These results demonstrate that the proposed methodology performs robustly across different domains, particularly excelling in summarizing core content and extracting meaningful relationships. This indicates its potential for broader applications in information retrieval, document classification, and technology analysis.

2) Quantitative evaluation metrics in existing research [7]

To compare the results of SAO-type labels generated by a probabilistic algorithm [Baseline] from an existing study [7] with those produced by an auto-labeling algorithm based on word embeddings [Title assignments with word embedding (TAWE)], which incorporates additional weights [15], and the results of the methodology proposed in this study, we utilize various metrics. Previous research has employed relevance, coverage, and discrimination metrics to select labels [7,15]. Each metric is defined as follows:

- Relevance: This measures the relevance of a final label $L_{i}$ to a specific topic $T_{i}$. It is calculated by averaging the absolute value of the cosine similarity between the vectors of each SAO structure s in $T_{i}\;and$ $L_{i}$.
- Coverage: This metric assesses how many words in SAO structure $S_{i}$ are represented by the final label${\;L}_{i}$ of topic $T_{i}$. It is calculated as the proportion of words in${\;L}_{i}$ that are present in the word set of $S_{i}$.
- Discrimination: This measures how distinct the final label $L_{i}$ of a particular topic $T_{i}$ is from the labels of other topics. It is calculated as the average absolute value of the cosine similarity between the label vectors of $T_{i}$ and all other topics, excluding itself.

The equations and notation for each metric are presented in Table 9.

**Table 9:** Relevance, coverage, discrimination equations and notation.

Index	Equation	Notation
Relevance	$\begin{array}{c} Relevance\left(T_{i}\text{,}L_{i}\right)=\frac{1}{{|S}_{i}|}\sum_{s{\in S}_{i}}CosineSimilarity\left(v_{L_{i}}\text{,}v_{s}\right) \end{array}$	$T_{i}:$ *The i − th topic* $L_{i}:$ *The final label of the i − th topic* $S_{i}:$ *The set of SAO (Subject−Action−Object) structures for topic i* $v_{L_{i}}:$ *Vector representation of the final label* $\;L_{i}$ $v_{s}:$ *Vector representation of the SAO structure sss* ${|S}_{i}|:$ *Number of SAO structures in topic i*
Coverage	$\begin{array}{c} Coverage\left(T_{i},\;L_{i}\right)=\frac{\left|{\{w\in S}_{i}|w\in \cup_{{s\in S}_{i}}s\}\right|}{\left|L_{i}\right|} \end{array}$	$T_{i}:The\;i-th\;topic$ $L_{i}:The\;final\;label\;of\;the\;i-th\;topic$ $S_{i}:The\;set\;of\;SAO\;(Subject-Action-Objectstructures\;for\;topic\;i$ $\cup_{{s\in S}_{i}}s:\;$ *The set of words from all SAO structures in topic i* $\left|L_{i}\right|:\;$ *The number of words in the final label* $L_{i}$
Discrimination	$\begin{array}{c} Discrimination\left(L_{i}\right)=1-\frac{1}{|T|-1}\sum_{T_{j}\in \;T,\;j\neq i}\left|CosineSimilarity(v_{L_{i}},v_{L_{j}}\right) \end{array}$	$T_{i}:The\;i-th\;topic$ $L_{i}:The\;final\;label\;of\;the\;i-th\;topic$ T: *The set of all topics* $v_{L_{i}}:$ *Vector representation of the final label* $L_{i}$ $v_{L_{j}}:$ *Vector representation of the final label* $L_{j}$ *|* T|: *The total number of topics*

The comparison results are shown in Tables 10 and 11, confirming that the methodology proposed in this study quantitatively outperforms previous approaches across both the AI and biotechnology domains.

**Table 10:** Comparison results based on the relevance, coverage, and discrimination metrics (AI).

	Topic 1		Topic 2		Topic 3		Topic 4		Topic 5		Topic 6		Topic 7	
Base Line [7]	Relev	0.0938	**Relev**	**0.1651**	Relev	0.0822	**Relev**	**0.1144**	Relev	0.1186	Relev	0.1038	Relev	0.1243
	Cover	0.0000	Cover	0.3333	Cover	0.3333	Cover	0.3333	**Cover**	**0.6667**	Cover	0.3333	Cover	0.0000
	Discri	0.8683	Discri	0.5321	Discri	0.5322	Discri	0.8888	Discri	0.7288	Discri	0.5325	**Discri**	**0.9369**
TAWE [15]	Relev	0.1236	Relev	0.1158	Relev	0.1055	Relev	0.1015	**Relev**	**0.128**	Relev	0.1395	Relev	0.086
	Cover	0.3333	Cover	0.6667	**Cover**	**0.6667**	Cover	0.3333	Cover	0.3333	Cover	0	Cover	0
	Discri	0.8811	Discri	0.8802	Discri	0.8486	Discri	0.8563	Discri	0.8697	Discri	0.8092	Discri	0.8777
Proposed	**Relev**	**0.1503**	Relev	0.1084	**Relev**	**0.1408**	Relev	0.1079	Relev	0.1073	**Relev**	**0.1526**	**Relev**	**0.1979**
	**Cover**	**0.6667**	Cover	0.3333	Cover	0.3333	**Cover**	**0.6667**	**Cover**	**0.6667**	**Cover**	**1.0000**	**Cover**	**1.0000**
	**Discri**	**0.8750**	**Discri**	**0.9048**	**Discri**	**0.8652**	**Discri**	**0.9054**	**Discri**	**0.9155**	**Discri**	**0.9131**	Discri	0.8313

**Table 11:** Comparison results based on the relevance, coverage, and discrimination metrics (Biotechnology).

	Topic 1		Topic 2		Topic 3	
Base Line [7]	**Relev**	**0.0955**	Relev	0.1401	Relev	0.1256
	**Cover**	**0.6667**	Cover	0.6667	Cover	0.6667
	Discri	0.8542	Discri	0.8598	Discri	0.8698
TAWE [15]	Relev	0.0931	Relev	0.1137	Relev	0.0855
	Cover	0.3333	**Cover**	**1.0000**	Cover	0.6667
	**Discri**	**0.9203**	Discri	0.9038	**Discri**	**0.9296**
Proposed	Relev	0.0882	**Relev**	**0.1786**	**Relev**	**0.1636**
	**Cover**	**0.6667**	**Cover**	**1.0000**	**Cover**	**1.0000**
	Discri	0.8586	**Discri**	**0.9121**	Discri	0.8916

In the AI domain, the proposed method achieved the highest average relevance score of 0.1380, outperforming the baseline score of 0.1146 and the TAWE score of 0.1122. This indicates that the SAO-based structure employed in this study allows the relevance score to more accurately reflect the core content of each topic. For coverage, the proposed method scored 0.6667 on average, which is significantly higher than the baseline’s 0.2857 and TAWE’s 0.3333, demonstrating its superior ability to capture key information from each topic. Regarding discrimination, the proposed method achieved a score of 0.8871, showing a marked improvement over the baseline score of 0.7171 and TAWE’s 0.8604, reflecting the positive impact of incorporating word embedding techniques to enhance label quality beyond what the original baseline approach provided. Similar trends were observed in the biotechnology domain. The proposed model showed the best performance in relevance, with an average score of 0.1435, exceeding the baseline’s 0.1204 and TAWE’s 0.0974, especially in Topics 2 and 3. In terms of coverage, both the baseline and TAWE models scored an average of 0.6667, while the proposed method achieved a notably higher average of 0.8889, indicating its effectiveness in capturing essential keywords. Discrimination also saw meaningful improvement, with the proposed model scoring 0.8874, which indicates that the method assigns clear and distinct labels while maintaining the uniqueness of each topic.

Taken together, these results demonstrate that the proposed labeling methodology consistently achieved higher performance across all three evaluation metrics in both domains. The results confirm that the proposed approach can clearly distinguish topic labels while preserving their semantic integrity. Overall, the methodology proposed in this study outperforms prior methods, particularly in the discrimination and coverage metrics. This indicates that the proposed approach is more effective at reflecting the core content of each topic and delineating topic boundaries, making it highly applicable to tasks such as information retrieval, document classification, and technology trend analysis.

### 4.5. Qualitative evaluation and interpretation of results

**Fig 10:**
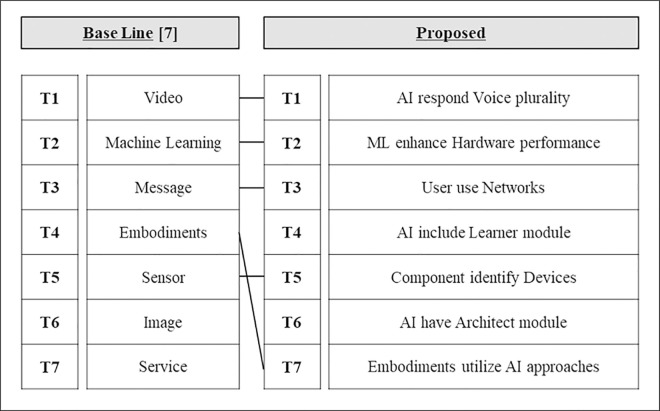
Similar topic relationships between the base line and proposed model classifications (AI).

**Fig 11:**
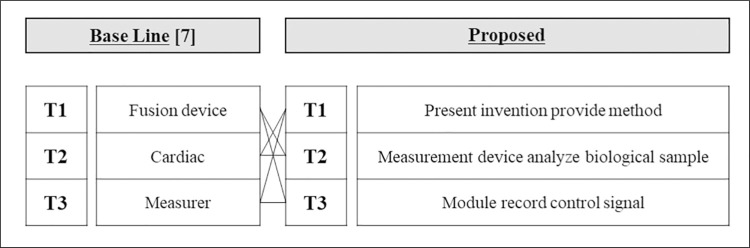
Similar topic relationships between the base line and proposed model classifications (Biotechnology).

While the quantitative evaluation methodology discussed in Section 4.4 is commonly used, this study highlights its contributions through a qualitative review and interpretation of the results, allowing for a more functional understanding of the technology as it relates to the SAO structure. We qualitatively compared these results with the existing word-level LDA outcomes and plotted the CPC-based hierarchy tree for further interpretation. The existing word-level auto-labeling LDA results from [7] are presented as the base line in Fig 10 and 11. To compare these with the labels generated by this study’s methodology (proposed), we connected them based on the word occurrences and semantic matches within the labels. This comparison reveals that our methodology offers more detailed functional information about the technology for three reasons:

1) It enables specific technical interpretation. Technical details are more clearly interpreted because topics and labels are organized according to each SAO element—subject, action, and object.

For example, in the existing methodology, the term “Machine Learning” is labeled as a simple phrase. In contrast, within the SAO structure, “Machine Learning” is categorized as a specific AI element, organized in a way that describes its function. This organization allows for a more nuanced interpretation of technical details.

2) Additionally, the SAO structure provides broader information coverage, as it describes conceptual units rather than individual words. This approach enhances contextual understanding and facilitates the analysis of interactions between topics. As illustrated in Figs 10 and 11, the proposed method allows for a more detailed description of patent content, including the extraction of functional information that is often overlooked by word-level models.
3) Furthermore, to validate the practical relevance of the results, expert opinions were collected from professionals in each technology domain. These experts evaluated the SAO-based topic labeling approach as highly effective in capturing both the functional aspects and contextual meaning of technologies. They also emphasized its usefulness for real-world applications such as technology trend analysis and R&D strategy development. This expert validation provides strong support for the practical applicability of the proposed methodology.

Thus, the proposed methodology offers more functional insights into technologies compared to traditional word-level LDA. This advancement is expected to contribute meaningfully to future technology trend predictions and industrial applications.

#### 4.5.1. AI Technology domain: Topic and CPC hierarchy insights.

Furthermore, as mentioned earlier, the H04 class was examined for the AI technology domain. The number and description of the CPC subclass distribution for a total of 700 patents are presented in Table 12.

**Table 12:** Description of [H04] class label.

Section	Class	Subclass	Count
H [ELECTRICITY]	H04 [ELECTRIC COMMUNICATION TECHNIQUE]	H04L [TRANSMISSION OF DIGITAL INFORMATION]	465
		H04N [PICTORIAL COMMUNICATION]	159
		H04W [WIRELESS COMMUNICATION NETWORKS]	110
		H04M [TELEPHONIC COMMUNICATION]	72
		H04B [TRANSMISSION]	31
		H04R [LOUDSPEAKERS, MICROPHONES, GRAMOPHONE PICK-UPS OR LIKE ACOUSTIC ELECTROMECHANICAL TRANSDUCERS; DEAF-AID SETS; PUBLIC ADDRESS SYSTEMS]	29
		H04S [STEREOPHONIC SYSTEMS]	6

**Fig 12:**
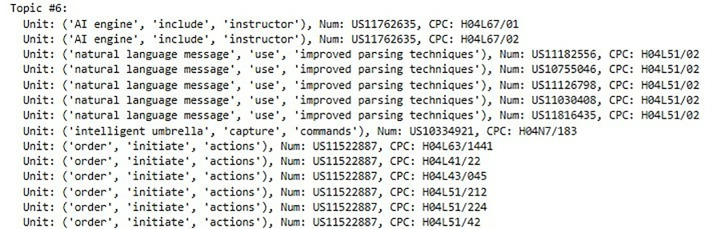
Examples of CPC subclass count by SAO unit (AI).

**Fig 13:**
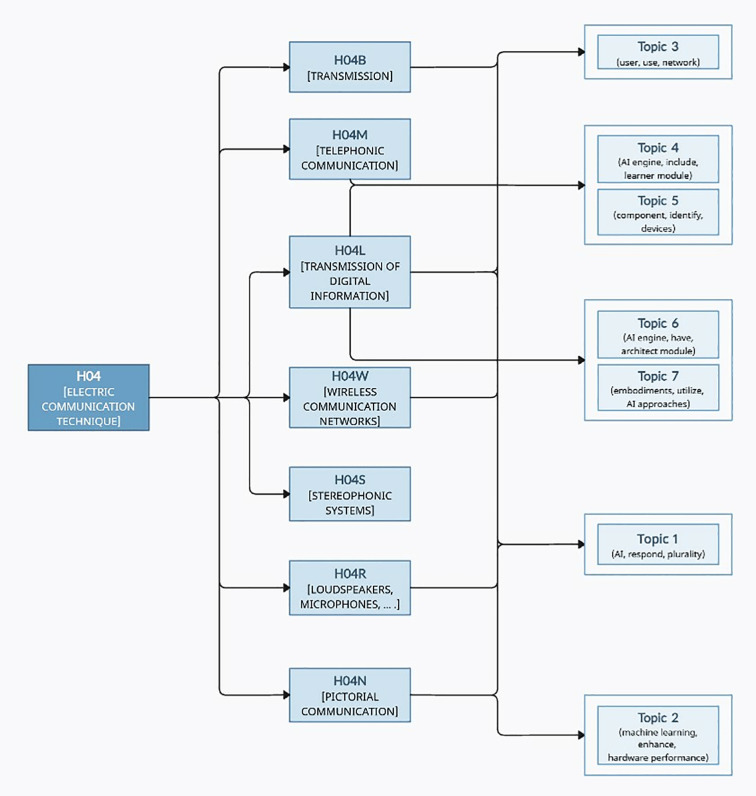
H04 class hierarchy tree.

At this point, the SAO units for each topic were cross-checked with the CPC subclasses of the extracted patents, as shown in Fig 12, and their occurrences were counted. Based on the frequency-based matching between SAO units and CPC subclasses, the hierarchical structure depicted in Fig 13 was constructed.

Topic 1 is labeled (AI, respond, plurality). The SAO units confirm that this topic appropriately describes an AI system interpreting and responding to computer commands. It is related to CPC subclasses H04N (interaction and computing environments) and H04L (digital communications). According to expert feedback, this topic effectively captures the direction of human-machine interaction technologies, particularly those central to recent AI-based digital assistants and interface technologies, supporting the high appropriateness of the assigned label.

Topic 2 is labeled (machine learning, enhance, hardware performance), highlighting the importance of the computing environment and AI-related components such as machine learning. The label emphasizes improvements in hardware performance and power efficiency, aligning with CPC subclass H04N. Experts note that this topic aligns well with the evolution of AI computing architectures (e.g., neuromorphic computing) and current industry focus areas like edge AI and low-power learning, confirming a strong correspondence between the label and the underlying technologies.

Topic 3 is labeled (user, use, network), focusing on technologies involving interactions between users, networks, and network operators. This topic corresponds to CPC subclasses H04L (digital communications), H04B (transmission technologies), and H04W (wireless communication networks). Experts observe that this topic fits the megatrend of communication-intelligence convergence, highlighting AI applications in 5G environments, thereby affirming the label’s apt summary of the topic’s functional content.

Topic 4 is labeled (AI engine, include, learner module), emphasizing AI engines, learning modules, and intelligent systems. The internalization of learning modules is regarded as a key element for self-learning AI systems requiring real-time adaptation, consistent with the technological flow highlighted in this topic. The relevant CPC subclasses are H04M (intelligent systems) and H04L. Expert opinions state that this label accurately reflects the AI system’s evolutionary trend toward enhanced autonomy.

Topic 5 is labeled (component, identify, devices), primarily describing component and device identification and data processing. It relates to CPC subclasses H04M and H04L. Experts highlight that the SAO units correspond closely to technologies actively used in IoT security, user authentication, and edge computing-based authentication systems, underscoring the label’s alignment with the primary technological objectives.

Topic 6 is labeled (AI engine, have, architect module), centering on architect modules, data collection, and conceptual node generation. It corresponds to CPC subclass H04L. Experts point out that the architect module’s process of constructing conceptual nodes is a core function in AI system architectures such as knowledge graph design and semantic classifiers, noting that both the SAO units and label effectively describe the technological direction and structural characteristics.

Topic 7 is labeled (embodiments, utilize, AI approaches), focusing on various embodiments—such as machine learning, AI engines, and user interfaces—that utilize AI approaches. It belongs to CPC subclass H04L. Experts confirm that the integration of diverse technologies within the SAO units reflects practical implementation scenarios in multimodal AI systems or agent-based automation systems. Considering this comprehensive technological integration, they state that the label suitably and broadly summarizes the technical content of the topic.

Interpreting these results, it is evident that recent research trends in the AI domain are predominantly classified under H04L, which can be attributed to the skewed CPC subclass distribution toward H04L, as illustrated in Table 12. As H04L addresses technologies related to the transmission, processing, and communication of digital information. It reflects the increasing importance of efficient information delivery and network-based interoperability alongside the advancement of AI technologies. According to expert opinions, recent research and development in the AI field have particularly focused on large-scale data transmission, real-time information processing, and network-based distributed AI systems. These trends play a critical role in various advanced application areas, including autonomous driving, smart cities, and multimodal AI. Therefore, the SAO-based analytical approach proposed in this study is useful for more clearly identifying the functional linkages and developmental trajectories of technologies within the AI domain. It is significant in that it enables detailed structural interpretations that reflect the complexity and interactions of these technologies.

#### 4.5.2. Biotechnology domain: Topic and CPC hierarchy insights.

In the case of the biotechnology domain, CPC Class A61 was reviewed. The distribution and descriptions of CPC subclasses across 3,209 patents are presented in Table 13.

**Table 13:** Description of [A61] Class Label.

Section	Class	Subclass	Count
A [HUMAN NECESSITIES]	A61 [MEDICAL OR VETERINARY SCIENCE; HYGIENE]	A61B [DIAGNOSIS; SURGERY; IDENTIFICATION]	1,958
		A61M [DEVICES FOR INTRODUCING MEDIA INTO, OR ONTO, THE BODY]	361
		A61K [PREPARATIONS FOR MEDICAL, DENTAL OR TOILETRY PURPOSES]	271
		A61N [ELECTROTHERAPY; MAGNETOTHERAPY; RADIATION THERAPY; ULTRASOUND THERAPY]	247
		A61F [FILTERS IMPLANTABLE INTO BLOOD VESSELS; PROSTHESES; DEVICES PROVIDING PATENCY TO, OR PREVENTING COLLAPSING OF, TUBULAR STRUCTURES OF THE BODY]	237
		A61L [METHODS OR APPARATUS FOR STERILISING MATERIALS OR OBJECTS IN GENERAL]	169
		A61P [SPECIFIC THERAPEUTIC ACTIVITY OF CHEMICAL COMPOUNDS OR MEDICINAL PREPARATIONS]	121
		A61H [PHYSICAL THERAPY APPARATUS]	79
		A61J [CONTAINERS SPECIALLY ADAPTED FOR MEDICAL OR PHARMACEUTICAL PURPOSES]	32
		A61C [DENTISTRY; APPARATUS OR METHODS FOR ORAL OR DENTAL HYGIENE]	23
		A61G [TRANSPORT, PERSONAL CONVEYANCES, OR ACCOMMODATION SPECIALLY ADAPTED FOR PATIENTS OR DISABLED PERSONS]	22
		A61Q [SPECIFIC USE OF COSMETICS OR SIMILAR TOILETRY PREPARATIONS]	11
		A61D [VETERINARY INSTRUMENTS, IMPLEMENTS, TOOLS, OR METHODS]	8

**Fig 14:**
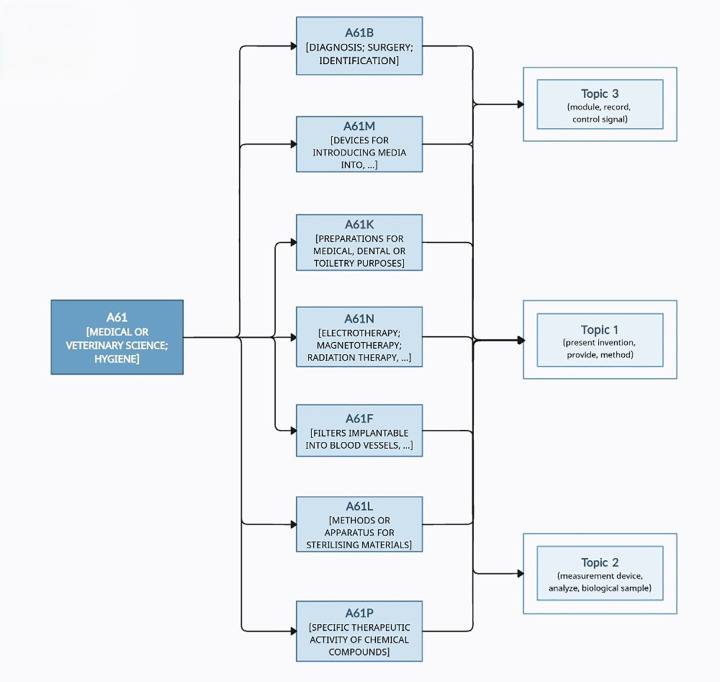
A61 class hierarchy tree.

As in the previous validation and interpretation process, CPC subclasses were counted with overlaps allowed, and based on their frequencies, a matching process was conducted. The resulting hierarchical structure of the CPC subclasses is shown in Fig 14, which highlights only the most meaningful subclasses.

The interpretations of each topic are as follows: Topic 1 is labeled (present invention, provide, method). The SAO units appropriately describe methods for implementing inventions related to various medical and diagnostic device components. This includes elements such as sensor connection methods, control mechanisms, and medical device design, capturing the core technical details close to actual implementations. The topic corresponds to CPC subclasses A61B (diagnosis; surgery; identification), A61M (devices for introducing media into or onto the body), A61K (preparations for medical, dental, or toiletry purposes), A61P (specific therapeutic activity of chemical compounds or medicinal preparations), and A61L (methods or apparatus for sterilising materials or objects in general). Expert feedback indicates that this topic effectively explains the core operational mechanisms of the invention and confirms the high appropriateness of the assigned SAO label.

Topic 2 is labeled (measurement device, analyze, biological sample, focusing on technologies that measure and analyze biological samples. The topic centers on the extraction of biological information and the detection of diseases or pathogens, providing a detailed functional explanation of the target analysis technology. Corresponding CPC subclasses include A61N (electrotherapy; magnetotherapy; radiation therapy; ultrasound therapy), A61B, and A61F (filters implantable into blood vessels). According to experts, the label “biological sample” is a highly relevant, comprehensive representation of the functional purpose of the topic, as it effectively reflects the interpretation and diagnostic use of biological information, affirming the strong suitability of the assigned SAO label.

Topic 3 is labeled (module, record, control signal), emphasizing modules within medical or diagnostic devices that record and process various control or biological signals. This topic belongs to CPC subclasses A61B and A61M. Experts note that the topic focuses on data collection and control execution functionalities, and the label appropriately reflects the control mechanism-based operation, demonstrating strong technical coherence and semantic consistency.

The analysis reveals that recent research trends in the Biotechnology field are largely concentrated under the A61B class across the three topics, which, similar to previous technical cases, can be attributed to the overall subclass distribution being skewed toward A61B. A61B is a CPC subclass that encompasses sensor-based diagnostic devices, signal recording apparatuses, and medical interface systems. This indicates that current technological development in Biotechnology is actively centered around diagnostic, recording, and analytical devices. According to expert opinions, this technological trend aligns with a broader industrial shift in biotechnology patents from a traditional focus on therapeutics to an increasing emphasis on diagnostics. While patents related to therapeutic development were previously considered highly valuable, the differentiation of such technologies has diminished over time. In contrast, diagnostic technologies can be applied widely regardless of the presence of disease, thus attracting greater attention from both industrial and strategic patent perspectives. As a result, many pharmaceutical and medical device companies are now intensifying their focus on diagnostic technologies, with some even establishing dedicated diagnostic divisions.

The SAO-based structural analysis method proposed in this study is shown to be an effective approach for capturing such technological transitions and flows. Specifically, in the biotechnology domain, this method successfully revealed the organic linkage among structural mechanisms, target entities, and control flows, enabling a detailed understanding of how diagnostic technologies operate and how they aim to solve specific problems.

In summary, a comparative review of its application to both the AI and Biotechnology domains shows that conventional classification or interpretation based solely on CPC codes is limited in identifying the operational principles and functional relationships within technologies. The SAO-based topic modeling and labeling approach proposed in this study enables a more functional understanding of technologies by interpreting patent document content through a Subject–Action–Object structure. This approach clarifies not only what a technology is intended to solve but also how it performs its functions.

By going beyond simple classification, it allows for deeper insights into the intrinsic functional structure of technologies. Researchers can thus gain a more comprehensive and structured understanding of the technical and functional aspects of patent documents, which is essential for identifying emerging technologies and trends. Furthermore, this function-oriented analytical approach enhances understanding of the purpose, execution, and problem-solving mechanisms of technologies compared to traditional keyword-based analyses. It holds strong potential for application in a wide range of industrial fields such as technology management, forecasting, and R&D planning—and is particularly well-suited for higher-level applications like problem-driven technology exploration, core technology identification, and technology convergence analysis.

## 5. Discussion

This section discusses the findings, limitations, and suggestions for improvement of the study. Section 2 provided a review of the literature on auto-labeling in topic modeling and explored the mathematical structure of SAO-based topic modeling. Sections 3 and 4 extended this by (1) identifying meaningful labels in technology development and industry practices, and (2) proposing a scoring-based auto-labeling methodology. This methodology applied text summarization and network analysis to SAO-based topic modeling results. The Silhouette score was employed to determine the appropriate k-means algorithm and identify the number of candidate labels, contributing to more accurate label extraction. Additionally, to validate the proposed model, (3) a quantitative verification was conducted using the ROUGE score to evaluate summary results, as well as relevance, coverage, and discrimination metrics from previous studies. The study further utilized the SAO structure, which highlights the functional importance of technology rather than relying solely on summarization. A qualitative validation was also introduced by comparing this approach with existing methods and generating a CPC subclass-based hierarchy tree. This enabled auto-labeling in topic modeling by integrating technology-specific information and resulted in a topic modeling method specialized for technology management.

To account for the technology level and hierarchy, a qualitative analysis using the patent CPC subclass was conducted after modeling. This approach offers an improved interpretation over traditional CPC code-based techniques, providing a deeper understanding of patent data and extracting various types of information. Consequently, researchers can achieve a more comprehensive view of emerging technologies and trends.

Despite these contributions, there are areas for improvement. To enhance the study’s results, more structured pattern definitions for SAO structure extraction may be required. While this study primarily focused on capturing the overall semantic relationships within SAO structures and applying an auto-labeling technique based on them, the systematic definition and analysis of specific patterns—such as problem–solution relationships—remain an important task for future research. For instance, incorporating structured patterns like (1) Problem & Solution, (2) Problem-only, and (3) Solution-only patterns could further refine the extraction and interpretation of SAO units. Such refinements are expected to improve the model’s interpretability and precision, thereby contributing to a deeper understanding of the technological innovation process.

There are also limitations related to the application domains. The study focused on “Artificial Intelligence” and “Biotechnology,” and additional illustrations from other domains may be necessary to further generalize the methodology and the interpretation of its results.

## 6. Conclusions

This study introduces a topic modeling and labeling method using the SAO structure, designed to specialize in identifying technology concepts and predicting technology trends. It proposes the use of text summary evaluation metrics along with existing auto-labeling metrics to assess model performance. Additionally, the study presents a hierarchy tree interpretation method based on patent CPC subclasses, which reflects the hierarchy structure of technologies. This approach provides researchers with functional insights when analyzing gaps between science and technology, making meaningful contributions to industrial applications. From a broader perspective, the study presents a novel topic modeling and labeling methodology tailored for patent documents in the field of technology management. Unlike traditional word frequency-based modeling, this approach incorporates semantic information, offering researchers deeper and more comprehensive insights. This is expected to help users quickly understand the topics by clarifying the core concepts of the technology and increasing the interpretability of each topic. In addition, unlike the existing methods, which are domain-specific and time-consuming due to the limitations of manual labelling by researchers or experts, this study uses the SAO structure to implement the automated labelling methodology more consistently. This can be considered as a contribution to reducing the time required for labelling and minimizing the variability due to subjective interpretation. The framework established in this study evaluates model performance from multiple dimensions, integrating quantitative metrics for text summarization with indicators that assess the semantic connectivity of technologies. By utilizing the patent CPC subclass, the study enhances the structural and semantic analysis of patent data, ultimately proposing a methodology for interpreting the results in alignment with the hierarchical structure of technology. This leads to meaningful findings that help researchers better understand the technical details of patents and provide a comprehensive understanding of emerging technological trends.

However, there are areas for improvement in the proposed model. First, the evaluation metrics used—ROUGE score, and the relevance, coverage, and discrimination metrics—may not be sufficient to fully assess all aspects of model performance. This limitation suggests the need for further research. To address this, future studies could incorporate structured, indicator-based qualitative evaluations such as expert validation, and simultaneously introduce advanced semantic coherence metrics capable of assessing deep semantic connections and topic consistency. These enhancements would diversify the evaluation framework and improve the robustness of the validation process. Second, there are limitations related to the application domains that can be addressed to enhance study outcomes. Generalizing the proposed process by applying it to other keyword fields could enable adaptation to various industries. In particular, in complex technological fields such as healthcare and biotechnology, semiconductors, robotics, and energy—where intricate interactions among components are critical—the SAO-based structural interpretation can effectively identify functional linkages between technologies and systematically track problem-solution oriented innovation flows. Finally, there are limitations related to the model itself. Future research may integrate Transformer-based deep learning techniques (e.g., BERT, T5) to improve the accuracy of SAO extraction and automate labeling processes further. Additionally, extending the methodology by employing Graph Neural Network (GNN)-based approaches to learn semantic relationships between SAO units could enhance overall analytical performance and interpretability.

## Supporting information

S1 Appendix Results of SAO-Based LDA and Labeling (AI). (DOCX)

S2 Appendix Results of SAO-Based LDA and Labeling (Biotechnology). (DOCX)
