# Supplementary material for: Developing Automatic-Labeled Topic Modeling Based on SAO Structure for Technology Analysis
Source: PLoS One. 2025 Aug 26;20(8):e0330275. doi: 10.1371/journal.pone.0330275 (PMC12380334; doi:10.1371/journal.pone.0330275)
Supplement: S1 Appendix — (DOCX) [file pone.0330275.s001.docx]

**S1 Appendix. Results of SAO-Based LDA and Labeling (AI).**

| **Topic 1**  (AI, respond, plurality) | **Topic 2**  (machine learning, enhance, hardware performance) | **Topic 3**  (user, use, network) | **Topic 4**  (AI engine, include, learner module) |
| --- | --- | --- | --- |
| ('clustering module', 'cluster', 'data values'): 0.2369  ('AI', 'respond', 'other commands'): 0.2031  ('determination', 'exceed', 'first parameter'): 0.1974  ('plurality', 'set', 'information'): 0.1001  ('order', 'deliver', 'end solution'): 0.0938 | ('system', 'include', 'cognitive agents'): 0.2001  ('AI engine', 'include', 'learner'): 0.1849  ('clustering module', 'identify', 'data fields'): 0.1693  ('machine learning', 'enhance', 'power consumption'): 0.1558  ('machine learning', 'enhance', 'hardware performance'): 0.1444 | ('nodes', 'be connected in', 'graph'): 0.1971  ('Network operators', 'use', 'algorithm'): 0.1760  ('user', 'use', 'network provider'): 0.1674  ('Network operators', 'use', 'operating parameters'): 0.1571  ('graph', 'be clustered into', 'peer groups'): 0.1489 | ('system', 'include', 'control decision'): 0.2003  ('system', 'include', 'learning engine'): 0.1984  ('clustering module', 'use', 'machine - learning models'): 0.1865  ('AI engine', 'include', 'architect module'): 0.1773  ('AI engine', 'include', 'predictor modules'): 0.1644 |
| **Topic 5**  (component, identify, devices) | **Topic 6**  (AI engine, have, architect module) | **Topic 7**  (embodiments, utilize, AI approaches) | **-** |
| ('component', 'identify', 'users'): 0.2308  ('privacy protection component', 'display', 'input data'): 0.2284  ('digital content', 'be linked to', 'location coordinates'): 0.2230  ('component', 'send', 'external communication'): 0.2143  ('user interface module', 'display', 'input data'): 0.2127 | ('architect module', 'create', 'concept node'): 0.2641  ('architect module', 'create', 'first concept node'): 0.2420  ('ingestion module', 'collect', 'input data'): 0.2215  ('AI engine', 'include', 'instructor'): 0.1834  ('aliasing module', 'apply', 'alias transform'): 0.1511 | ('machine learning hardware', 'perform', 'distribution'): 0.2495  ('AI engine', 'be beclosed with', 'plurality'): 0.2094  ('machine learning hardware', 'have', 'DNN module'): 0.1283  ('named entities', 'use', 'reduction rules'): 0.1148  ('embodiments', 'utilize', 'network graph approach'): 0.0928 | **-** |
