# Supplementary material for: Developing Automatic-Labeled Topic Modeling Based on SAO Structure for Technology Analysis
Source: PLoS One. 2025 Aug 26;20(8):e0330275. doi: 10.1371/journal.pone.0330275 (PMC12380334; doi:10.1371/journal.pone.0330275)
Supplement: S2 Appendix — (DOCX) [file pone.0330275.s002.docx]

**S2 Appendix. Results of SAO-Based LDA and Labeling (Biotechnology).**

| **Topic 1**  (present invention, provide, method) | **Topic 2**  (measurement device, analyze, biological sample) | **Topic 3**  (module, record, control signal) | - |
| --- | --- | --- | --- |
| ('controller', 'connect', 'analyte measurer'): 0.4015  ('spectrometer', 'include', 'plurality'): 0.3493  ('present invention', 'provide', 'novel medical instrument'): 0.3424  ('interconnecting member', 'stabilize', 'IVIs'): 0.3297  ('MMTD', 'comprise', 'cardiac signal circuit'): 0.2372 | ('gene', 'be expressed in', 'biological sample'): 0.3487  ('corresponding signal', 'be induced within', 'external element'): 0.3030  ('measurement device', 'include', 'sensor'): 0.2958  ('order', 'measure', 'blood characteristics'): 0.2834  ('device', 'provide', 'robust timing mechanism'): 0.1384 | ('recorder', 'record', 'motion measurer'): 0.4115  ('biosensor', 'include', 'reagent'): 0.3441  ('controller', 'include', 'damage determiner'): 0.2955  ('MP', 'transmit', 'control signal'): 0.2761  ('controller', 'connect', 'recorder'): 0.1734 | - |
